# Measurement of Shoulder Range of Motion in Patients with Adhesive Capsulitis Using a Kinect

**DOI:** 10.1371/journal.pone.0129398

**Published:** 2015-06-24

**Authors:** Seung Hak Lee, Chiyul Yoon, Sun Gun Chung, Hee Chan Kim, Youngbin Kwak, Hee-won Park, Keewon Kim

**Affiliations:** 1 Department of Rehabilitation Medicine, Seoul National University Hospital, Seoul, Korea; 2 Interdisciplinary Program of Bioengineering, Seoul National University Graduate School, Seoul, Korea; 3 Institute of Medical and Biological Engineering, Medical Research Center and Department of Biomedical Engineering, Seoul National University College of Medicine, Seoul, Korea; 4 Department of Rehabilitation Medicine, Gangwon-do Rehabilitation Hospital, Gangwon-do, Korea; University of Szeged, HUNGARY

## Abstract

Range of motion (ROM) measurements are essential for the evaluation for and diagnosis of adhesive capsulitis of the shoulder (AC). However, taking these measurements using a goniometer is inconvenient and sometimes unreliable. The Kinect (Microsoft, Seattle, WA, USA) is gaining attention as a new motion detecting device that is nonintrusive and easy to implement. This study aimed to apply Kinect to measure shoulder ROM in AC; we evaluated its validity by calculating the agreement of the measurements obtained using Kinect with those obtained using goniometer and assessed its utility for the diagnosis of AC. Both shoulders of 15 healthy volunteers and affected shoulders of 12 patients with AC were included in the study. The passive and active ROM of each were measured with a goniometer for flexion, abduction, and external rotation. Their active shoulder motions for each direction were again captured using Kinect and the ROM values were calculated. The agreement between the two measurements was tested with the intraclass correlation coefficient (ICC). Diagnostic performance using the Kinect ROM was evaluated with Cohen’s kappa value. The cutoff values of the limited ROM were determined in the following ways: the same as passive ROM values, reflecting the mean difference, and based on receiver operating characteristic curves. The ICC for flexion/abduction/external rotation between goniometric passive ROM and the Kinect ROM were 0.906/0.942/0.911, while those between active ROMs and the Kinect ROMs were 0.864/0.932/0.925. Cohen’s kappa values were 0.88, 0.88, and 1.0 with the cutoff values in the order above. Measurements of the shoulder ROM using Kinect show excellent agreement with those taken using a goniometer. These results indicate that the Kinect can be used to measure shoulder ROM and to diagnose AC as an alternative to goniometer.

## Introduction

Adhesive capsulitis of the shoulder (AC), also known as “frozen shoulder,” is a common shoulder disorder in middle age with an estimated prevalence of 2% in the general population [1]. In principle, AC is clinically diagnosed based on history and physical examination, when shoulder range of motion (ROM) is limited in all directions without structural lesion [2, 3]. Thus, measurement of the shoulder ROM is important for the diagnosis of AC as well as the follow-up evaluation. ROM is usually measured with a goniometer; however, conventional goniometric measurements can vary among testers [4] and require substantial time and effort in clinics.

Recently, the Kinect markerless three-dimensional (3D) depth camera was developed as an interface device and has two versions, one for the Xbox 360 home entertainment system and another for Windows applications. The Kinect obtains a depth image by recognizing the pattern of the infrared points emitted by the projector within it. The depth image is processed to provide coordinates of predefined body segments. This new device enables real-time, 3D motion capture in an easy-to-implement and nonintrusive way.

There have been several attempts to apply the Kinect for Windows to medicine such as smart home tele-rehab settings for stroke patients [5] and therapeutic games for children with cerebral palsy [6, 7]. In one study, reachable workspace of the upper extremity, which involves ROM of the shoulder, was assessed with the Kinect in patients with neuromuscular disease [8]. Some studies compared shoulder ROM measurements using Kinect with those by motion capture system or goniometer in healthy population [9, 10]. However, it has not been proven that the Kinect provides enough accuracy to enable its use in clinical assessment.

In this study, we hypothesized that the Kinect for Windows can be used to measure the shoulder ROM of patients with AC. To verify this, we first compared the shoulder ROM values measured with using Kinect with those measured using a conventional goniometer under optimal conditions. Second, we attempted diagnosing AC based on the Kinect ROM measurements.

## Materials and Methods

### 2.1 Participants

Fifteen healthy controls (HCs) and 12 patients with AC participated in the study. The HCs had never experienced any type of shoulder disease and volunteered to participate. AC was diagnosed if one’s passive ROM of the shoulder measured with a goniometer were limited in at least two directions and the limitation of motion (LOM) persisted for more than 1 month without radiological abnormalities. ROMs were determined as limited with a flexion <165°, abduction <150°, or external rotation <45°. Patients were excluded if they had a history of major trauma involving the shoulder, symptomatic rotator cuff tear evidenced by ultrasonography, inflammatory joint disease, or any other structural or systemic disorder accounting for shoulder pain or LOM. All of the patients underwent radiographic and ultrasonographic evaluation before enrollment. Fifteen HCs (45 ± 9 years old; eight men) and 12 AC patients (52 ± 9 years; six men) were recruited. Mean AC symptom duration in the patients was 17 ± 16.8 months (range, 2–60 months). All of the patients had unilateral AC (Table 1). The study protocol was approved by the research ethics board at Seoul National University Hospital and all participants gave written informed consent prior to enrollment.

**Table 1:** Subjects’ clinical characteristics.

	**HCs**	**Patients with AC**
****Number of subjects****	15	12
****Age (mean ± SD)****	45.47 ± 9.05	51.50 ± 9.04
****Sex (male/female)****	8/7	6/6
**Symptom duration (months, mean ± SD)**	-	17.00 ± 16.8
****Lesion side (right/left)****	-	6/6

HC, healthy controls; AC, adhesive capsulitis; SD, standard deviation

### 2.2 Goniometric measurement

**Fig 1:**
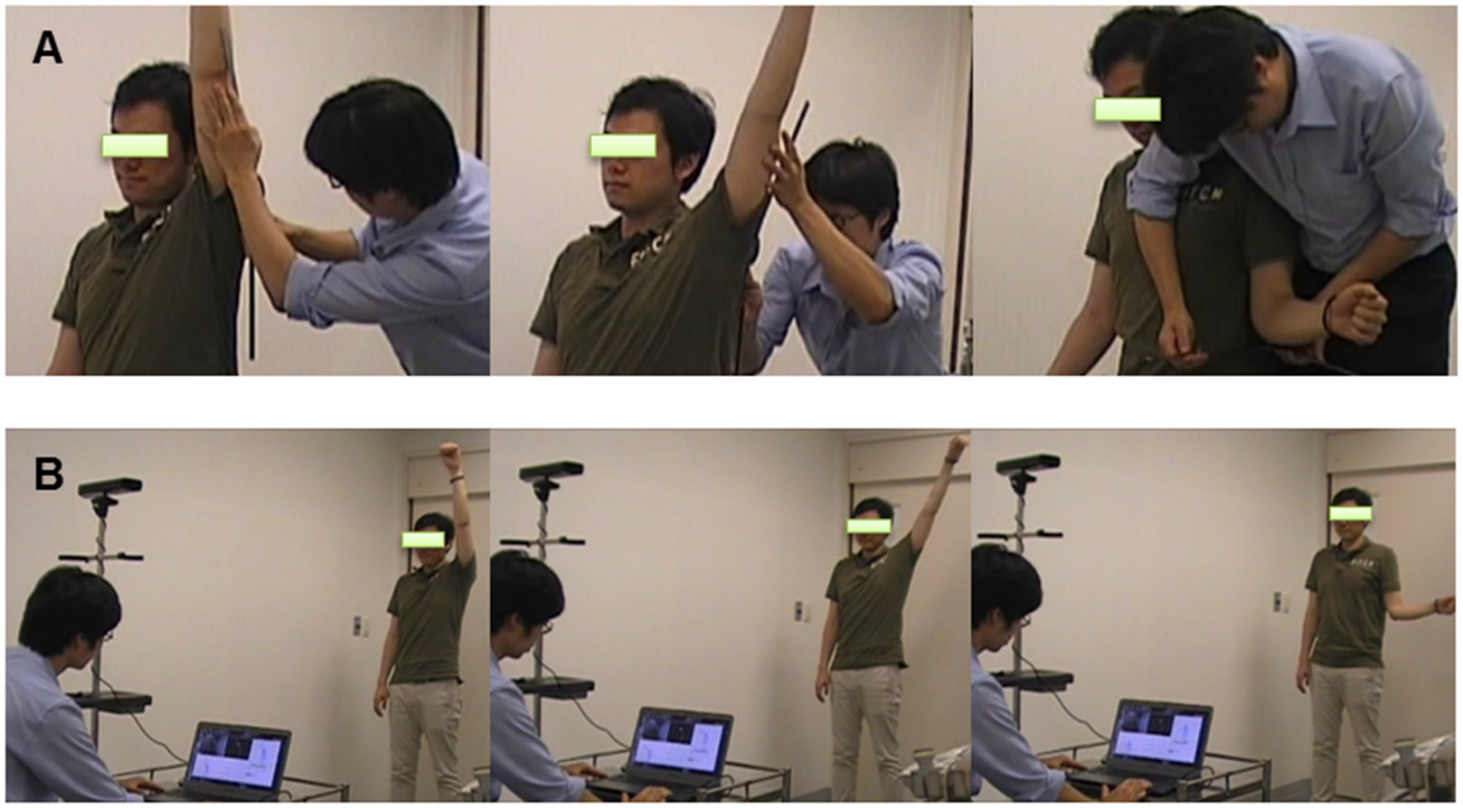
Measurement of the shoulder range of motion (ROM). A. Goniometric active ROM measurement of flexion, abduction, and external rotation by the examiner. B. The Kinect measurement of flexion, abduction, and external rotation ROM under instruction from the examiner. The individual in this figure has given written informed consent (as outlined in PLOS consent form) to publish these case details.

One examiner measured passive ROMs (pROMs) and active ROMs (aROMs) for flexion, abduction, and external rotation at a neutral position using a stainless steel goniometer (JAMAR Co., Pakistan). Each subject was seated on a stool and the examiner measured the ROM in each direction using a goniometer (Fig 1A). Scapular rotation was allowed for flexion and abduction. External rotation was measured in a neutral position with the shoulder adducted, the elbow flexed at the right angle, and the forearm in a neutral supination-pronation position; the angle between the long axis of the forearm and the sagittal plane of the trunk was determined as rotational ROM. The aROMs were measured under the instruction that subjects should move their arm as far as they could, while the pROMs were measured by the examiner moving each subject’s arm until limited mechanically or by pain.

### 2.3 Kinect measurement

A Kinect for Windows was placed at a fixed height of 1.7 m and the subjects stood at the distance of 2 m from it according to the Kinect instructions. Subjects were standing in front of the camera and only the frontal body surface was detectable during skeletal tracking. All subjects performed three active motions: shoulder flexion, abduction, and external rotation (Fig 1B). The direction for each motion was basically the same as described above. They were verbally instructed to move their arm in the plane as far as they could. They repeated each motion 3–5 times at a comfortable speed while standing. For the HCs, the ROM (passive, active, or with Kinect) of both shoulders were measured; for the patients with AC, the ROM of the affected shoulders were measured for the analysis.

### 2.4 Calculation of the shoulder ROM measured with the Kinect

**Fig 2:**
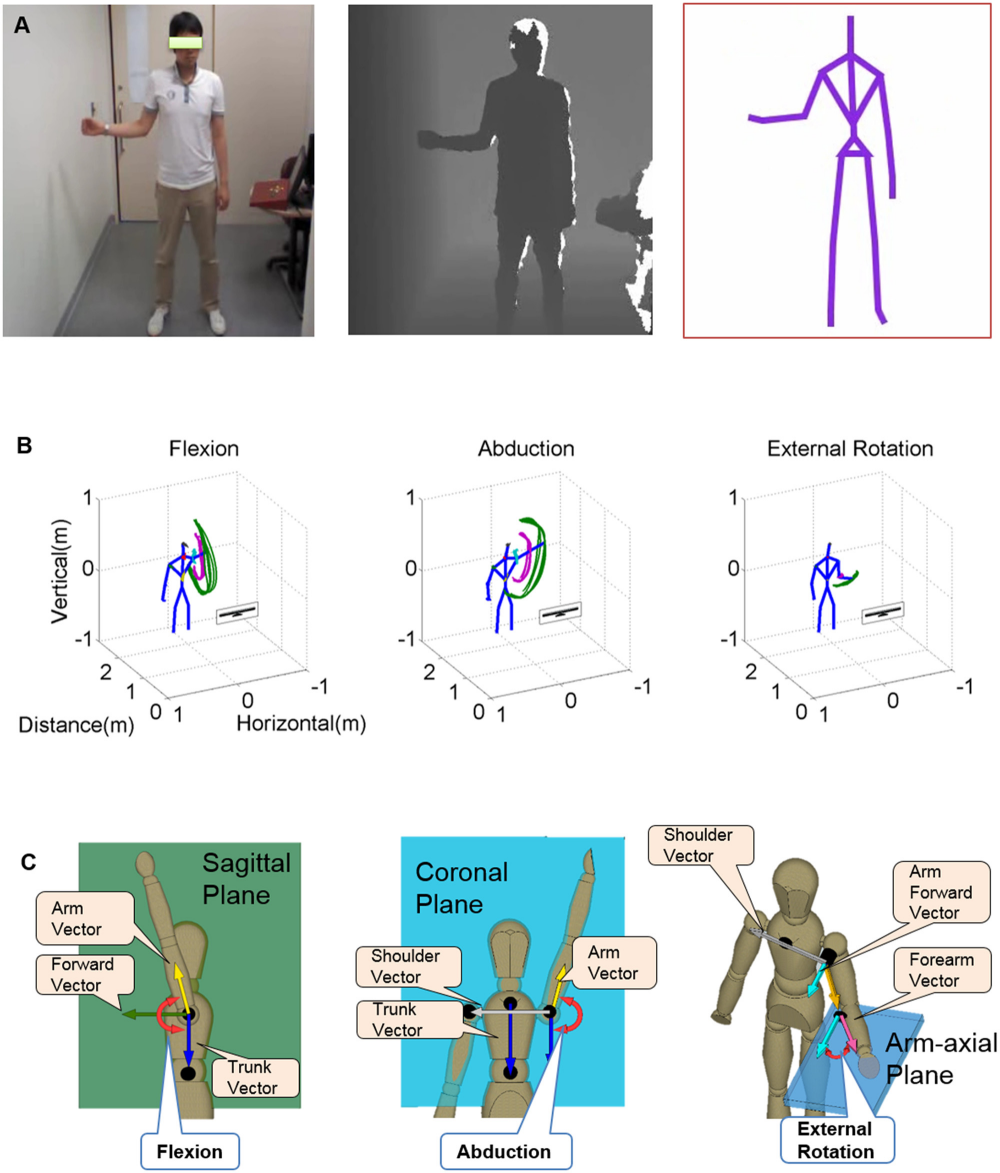
Measurement and calculation of the shoulder range of motions (ROMs) using the Kinect. A. Red, green, and blue image, depth image, and converted skeleton image from the Kinect. B. Traces of body segments during each shoulder motion. The green line is the trace of the left wrist, while the pink line is the trace of the left elbow. C. Calculation of the shoulder ROM angles by the projected angles on the defined anatomical planes. The individual in this figure has given written informed consent (as outlined in PLOS consent form) to publish these case details.

The Kinect continuously captures depth images (30 frames/s) and detects body segments. Kinect for Windows SDK 1.6 (Microsoft) converted them to real-time 3D coordinates of 20 body parts (Fig 2A and 2B). In this study, the coordinates of the head, neck (designated as “center of shoulders” in the MSDN Kinect for Windows library), umbilicus (designated as “spine”), shoulders, elbows, and wrists were used to calculate the shoulder ROM measured with the Kinect (kROMs) (Fig 2A and 2B).

For calculation of the kROMs, the anatomical planes of the body were defined as follows: the coronal plane was defined to include “the vector from the left shoulder to the right shoulder” (shoulder vector) and “the vector from the neck to the umbilicus” (trunk vector); the sagittal plane was defined to include the “trunk vector” and “the cross product of shoulder vector and trunk vector” (forward vector). Flexion angle of the shoulder was calculated as the projected angle on the sagittal plane between “the vector from the shoulder to the elbow” (arm vector) and the “trunk vector”; abduction angle of the shoulder was calculated as the projected angle on the coronal plane between the “arm vector” and the “trunk vector.” Rotational angle of the shoulder was calculated as the projected angle on “the plane perpendicular to the arm vector” (the arm-axial plane) between “the vector from the elbow to the wrist” (forearm vector) and “the cross product of the shoulder vector and the arm vector” (arm forward vector) (Fig 2C). The peak angles of each repeated motion were averaged to determine the ROM angle in that direction. All of the calculations were made with MATLAB (MATLAB2013a; Mathworks, Natick, USA).

### 2.5 Statistical Analysis

#### 2.5.1 Validity of Kinect to measure shoulder ROM

To test validity, we evaluated the agreement between kROMs and goniometric ROM measurements (aROMs, pROMs) using intraclass correlation coefficients (ICC) and Bland-Altman plots.

#### 2.5.2 Utility of Kinect measurement for the diagnosis of AC

To evaluate the clinical applicability of the Kinect measurement, the diagnostic yield of AC in the study participants was calculated using Cohen’s kappa values. The diagnostic criteria of the Kinect measurement for AC were basically the same as those of the goniometric passive ROM measurement: ROM should be limited in at least two directions, and the LOM had persisted more than 1 month without radiological abnormalities. The duration of LOM and radiological information were the same as the goniometric diagnosis. However, the cutoff values for LOM when measured with the Kinect were determined in three ways: the same as the goniometric passive ROM measurement; reflecting the mean difference between the goniometric measurement and the Kinect measurement; and optimal cutoff values inferred from receiver operating characteristic (ROC) curves. The significance of the comparison of kROMs between the HCs and the patients with AC was tested with the Mann-Whitney *U* test. P values <0.05 were statistically significant.

## Results

### 3.1 Agreement between goniometric and Kinect measurements

The aROMs measured with a goniometer and the kROMs showed excellent agreement for each direction (ICCs: flexion, 0.864; abduction, 0.932; external rotation, 0.925). The pROMs and kROMs showed excellent agreement in each direction as well (ICC: flexion, 0.906; abduction, 0.942; external rotation, 0.911). The agreement between the aROMs and pROMs was excellent as expected (ICC: flexion, 0.918; abduction, 0.992; external rotation, 0.965).

**Fig 3:**
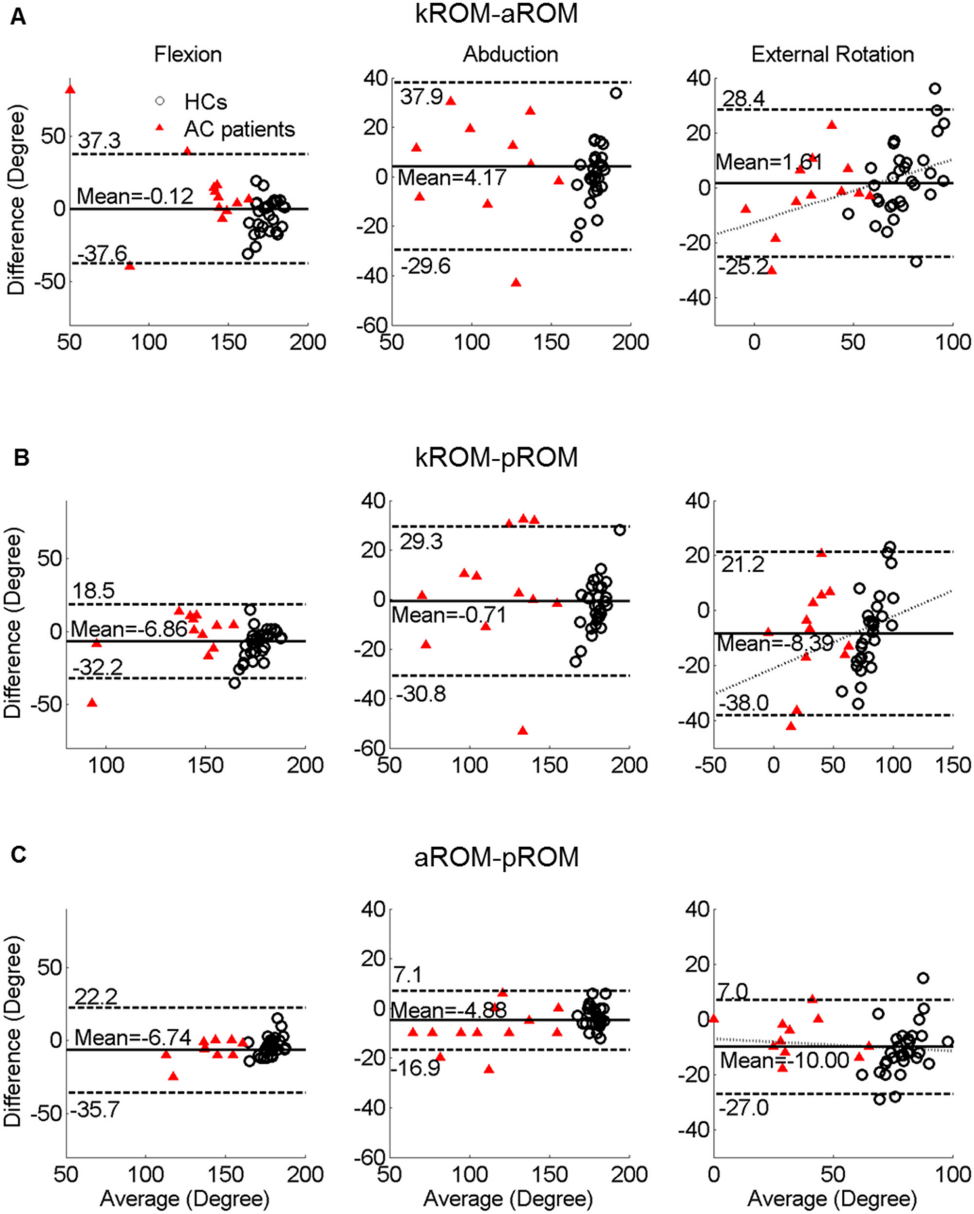
Bland-Altman plots of kROMs, pROMs, and aROMs. The circles show 30 shoulders of 15 healthy controls, while the triangles show 12 affected shoulders of patients with adhesive capsulitis (42 shoulders total). Mean differences are indicated by the solid line and 95% limits of agreement (mean differences ± 1.96 standard deviation of the difference) are shown by the dashed line. The dotted line shows regression lines for proportional biases. A. Comparison between kROMs and aROMs. B. Comparison between kROMs and pROMs. C. Comparison between aROMs and pROMs. kROMs, shoulder range of motion measured with the Kinect; pROMs, passive shoulder range of motion measured with the goniometer; aROMs, active shoulder range of motion measured with the goniometer.

Bland-Altman plots were drawn. The mean differences of the kROMs from the aROMs (kROMs–aROMs) were -0.12° (flexion), 4.17° (abduction), and 1.61° (external rotation). The limits of agreement determined by the 95% confidence interval were -37.6–37.3° for flexion, -29.6–37.9° for abduction, and -25.2–28.4° for external rotation (Fig 3A). The mean differences of the kROMs from the pROMs were -6.86° (flexion), -0.71° (abduction), and -8.39° (external rotation). The limits of agreement determined by the 95% confidence interval were -32.2–18.5° for flexion, -30.8–29.3° for abduction, and -38.0–21.2° for external rotation, respectively (Fig 3B).

### 3.2 Kinect measurement for patients with AC

**Fig 4:**
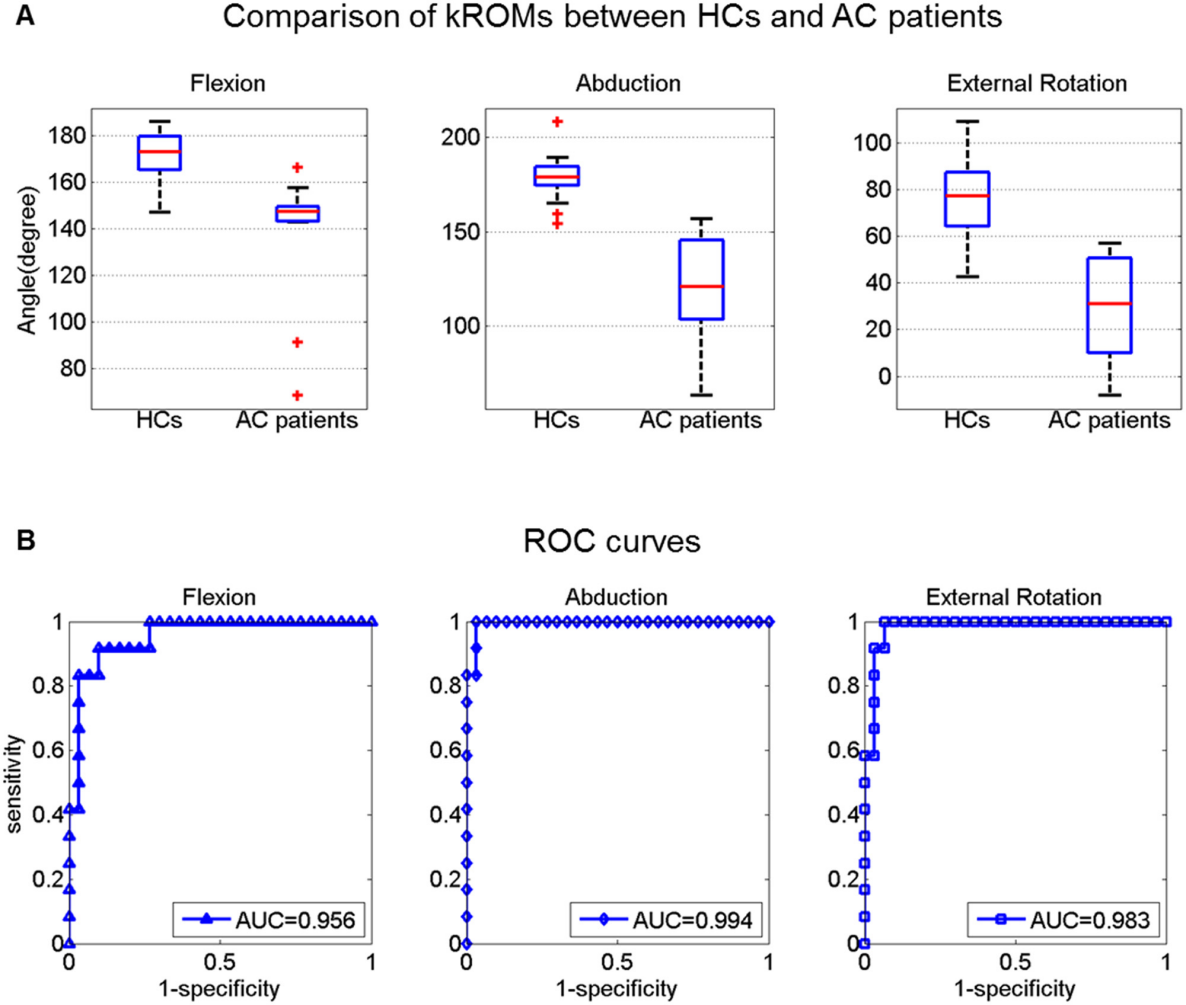
Diagnosis of adhesive capsulitis of the shoulder (AC) using shoulder range of motion measured with the Kinect (kROMs). A. Box plots for the comparison of kROMs between healthy controls (HCs) (N = 30) and AC patients (N = 12) for flexion, abduction, and external rotation, respectively. B. Receiver operating characteristic curves for the diagnosis of AC using kROMs in each direction. Areas under the curves are shown.

The utility of the Kinect for the diagnosis of AC was obtained based on three different cutoff values for limited ROMs. When the same cutoff values with those of the goniometric passive ROM (flexion <165°, abduction <150°, or external rotation <45°) were applied, 10 of 12 patients with AC were diagnosed with AC (Cohen’s kappa = 0.88). When the cutoff values were determined to reflect the mean differences of kROMs from pROMs (flexion <158.1°, abduction <149.3°, or external rotation <36.6°), 10 of 12 patients with AC were also diagnosed with AC (Cohen’s kappa = 0.88). ROC curves were drawn for each direction (Fig 4B). The areas under the curves were nearly 1 (>0.95) for all directions. When optimal cutoff values were determined from the ROC curves using the Youden index (flexion <158.3°, abduction <159.1°, or external rotation <59.1°), all of the patients with AC were diagnosed with AC (Cohen’s kappa = 1) (Table 2). None of the HCs were diagnosed with AC using any of the above cutoff values. The kROMs in the patients with AC were significantly smaller than those in the HCs for all of the directions (p = 0.000, 0.000, and 0.000 for flexion, abduction, and external rotation, respectively, with the Mann-Whitney U test) (Fig 4A).

**Table 2:** Diagnostic performance of the Kinect for adhesive capsulitis with different cutoff values.

Cutoff values for limited ROM	Cohen’skappa	Flexion		Abduction		External rotation	
		Se	Sp	Se	Sp	Se	Sp
**Same with pROM cutoff values** ^a^	0.88	0.92	0.80	0.75	1.00	0.58	0.97
**Reflecting mean differences** ^b^	0.88	0.92	0.90	0.67	1.00	0.50	1.00
**Optimal values from ROC curves** ^c^	1.00	0.92	0.90	1.00	0.97	1.00	0.93

ROM, range of motion; Se, sensitivity; Sp, specificity; pROM, passive ROM; ROC, receiver operating characteristic

^a^Adhesive capsulitis is diagnosed when ROM was limited in two or more directions. Cutoff values for limitation were defined in three ways: same with goniometric pROM cutoff values (flexion <165°, abduction <150°, or external rotation <45°).

^b^reflecting mean differences (flexion <158.1°, abduction <149.3°, or external rotation <36.6°).

^c^optimal values from ROC curves (flexion <158.3°, abduction < 159.1°, or external rotation <59.1°).

Cohen’s kappa values show the diagnostic performances of the Kinect measurement with each cutoff value.

## Discussion

### 4.1 Shoulder ROM measurements using the Kinect

In this study, we compared shoulder ROMs measured using a Kinect and those measured using a conventional goniometer. Additionally, we evaluated its applicability for the diagnosis of AC. Although there were measurement differences between two methods, ICC values in three cardinal directions (flexion, abduction, and external rotation) verified excellent agreement of Kinect measurements with goniometric measurements of shoulder ROMs. Furthermore, the Kinect was clinically applicable to detect limited shoulder ROMs, which was critical for diagnosing patients with AC.

### 4.2 Comparison with Previous Studies

There were a few previous studies that evaluated shoulder motions using Kinect. Nixon et al. assessed the accuracy of joint angle data determined with the Kinect in random upper extremity movements by comparison with the Vicon camera system (Vicon, Oxford, UK). They showed an average error of these angle measurements not exceeding 10.0% [9]. Hawi et al. adopted the Kinect for measuring the shoulder ROM and compared it with the goniometric measurement. According to their result, the Kinect measurements showed good test-retest reliability and poor–to-moderate agreement with the goniometric measurements [10]. However, their study did not specify in detail how they measured ROMs with a goniometer and as well as with Kinect. As mentioned earlier, measurements of shoulder ROM with a goniometer quite varies depending on how it was measured: whether scapular motion was controlled, how the plane was defined, or whether the ROM was active or passive. In addition, the experimental conditions with Kinect such as the locations of the camera were not explained, which could also influence the results [11]. There might be fixed or proportional biases between the two methods that could be reduced by modification of the experimental settings. Moreover, when compared with the current study, both studies recruited only healthy volunteers. It could not be determined whether the resultant accuracy of Kinect was clinically applicable or not. In our study, we conducted ROM measurements in a specified way. Accordingly, we could observe improved reliability and agreement enough to discern the patients with AC from the healthy controls.

### 4.3 Clinical benefits of Kinect

Although goniometry is not an objective method [4] and requires tester effort, it is the most commonly used method to assess the shoulder ROMs in clinics because of its simplicity [12]. Some studies have measured shoulder ROMs using an optical motion capture system to obtain more objective and accurate values [13]. This system has excellent accuracy, but it is very expensive and inconvenient to prepare, measure, and calculate angles, thereby undermining its clinical applicability.

To overcome the disadvantages of the motion capture system, new motion analysis hardware such as inertial sensors [14, 15] and Kinect [8, 16] has been introduced. These new devices are simpler. In particular, the Kinect has several advantages. First, the measurements taken with it are not tester-dependent; thus, it can be considered an objective as well as quantitative method [17]. Second, it is nonintrusive. Subjects do not need to wear or touch anything and testers do not need to hold or align anything; rather, subjects only have to move in front of a Kinect to have their ROM checked. Third, it is a low-cost and easy-to-implement device. The price of the Kinect for Windows is approximately 250 US dollars. The device is directly connected to a personal computer via USB and operates with a free download application, “Kinect for Windows SDK.”. Once it is placed in front of a person, it automatically detects the individual’s body segments and returns their coordinates in real time. Furthermore, if employed in tandem with communication technology such as the Internet, it may provide a new tele-medicine method.

### 4.4 Measurement differences between Kinect and goniometer

This study revealed the existence of systematic differences (fixed bias) between kROMs and pROMs, the standard parameter for the diagnosis of AC. The mean kROMs for flexion, abduction, and external rotation were smaller than the mean pROMs by <10° (Fig 3B). Additionally, the aROMs were significantly smaller than the pROMs in all directions (p = 0.000, 0.000, and 0.000 for flexion, abduction, and external rotation, respectively; Fig 3C). The magnitude of the difference was similar with that between the kROMs and pROMs. Those differences supposedly resulted from a general observation that passive motion extends beyond active motion with the help of a tester [18, 19]. Therefore, fixed bias between the kROMs and the pROMs would be attributed mainly to the difference of active motion from passive motion and it implies that Kinect measurement is more similar to goniometric aROM.measurement than pROM measurement.

Along with the systematic difference, the B-A plot for external rotation demonstrated a proportional bias of the kROMs for the pROMs and aROMs (Fig 3A and 3B). The regression line of the plot had a positive slope, which indicated that kROMs tended to be larger than aROMs or pROMs, when the extent of external rotation of the subject’s shoulders was more. It was presumed that the subjects slightly abducted their shoulders on external rotation during Kinect measurements. The subjects could abduct their shoulders to a greater degree in front of the Kinect because their movements were not controlled in a given plane by a tester. External rotation angle can be greater in a more abducted posture because anatomically, restraint by the ligaments against external rotation is less in the abducted posture [20].

The 95% limit of agreement for two measurements with each instrument ranged from −30° to 30° in all directions. This seems to be due to subjects’ unrestricted motions during the kROMs measurement, which is more likely to deviate from the planes. During kROMs measurements, verbal instructions were given to control each subject’s motion in each plane and the subjects could freely move their arm in a space without any mechanical restriction; that is, the motions were inevitably out of the anatomical planes. The goniometric measurements were more constrained in the planes guided by the tester’s measuring behavior. This limitation can be resolved via the development of a device that constrains a subject’s motion in each plane for the Kinect measurement. However, it can seriously affect the simplicity of using the Kinect. We have conducted a supplementary experiment to evaluate the difference between Kinect measurement and goniometric measurement while minimizing the effect of unrestricted movement which had been allowed during Kinect measurement. Seven healthy volunteers were further recruited and their both shoulders were assessed. The subjects were instructed to stand in front of the Kinect with a particular shoulder position within 3 cardinal planes. We measured the joint angle with a goniometer, and then Kinect in rapid succession while the subject maintained the position. The results showed remarkable decrease in 95% limit of agreement between two measurement methods in all directions. The mean differences of the measurements were 1.77° (flexion), -1.08° (abduction), and 1.19° (external rotation). 95% limits of agreement were -14.82–18.36° for flexion, -9.02–6.87° for abduction, and -10.89–13.26° for external rotation. These results indicate that the unrestricted motion of Kinect measurement was an important source of the increased variability.

Discrepancies between measurement postures can be a source of the difference. We performed a goniometric ROM measurement when the subjects were seated but Kinect ROM measurement was performed for the subjects in a standing position. It was inevitable because the camera setting was optimal for the standing position, and if the subjects are seated, skeletal tracking can be distorted. However, its impact seems to be insignificant because the coordination change of the umbilicus was not great during the standing measurements.

Besides, measurement errors during image capture with Kinect might be a source of the difference. However, when the reliability of the Kinect for the ROM measurement was assessed based on repeated motions during the study, the ROM values were strikingly consistent (ICC: flexion, 0.97; abduction, 0.98; external rotation, 0.98). Thus, the influence of the measurement error during image recording seems minimal.

Another possible source of the difference is the calculation method for ROM angles. We used the projected angle on each anatomical plane between the two corresponding vectors. This method is simple but does not reflect subjects’ motions that are outside the planes. If we employ a more complicated method of calculating ROM angles that considers 3D motion in an arbitrary direction, the difference may be avoided.

Of note, scapular motion was allowed in this study and we did not distinguish gleno-humeral motion from thoraco-humeral motion. In clinical practice, some physicians limit scapular movement and measure gleno-humeral ROMS with a goniometer while others do not. The difference is a source of variation among testers and Kinect cannot evaluate scapular motion as it is. For the comparison with measurement with Kinect in the current study, we did not consider scapular motions separately.

### 4.5 Cutoff values for ROM limitation in patients with AC

The cutoff values for the LOM of the shoulder to diagnose AC in this study (flexion <165°, abduction <150°, or external rotation <45°) were relatively higher than those of other studies [21, 22]. The patients in this study were in diverse phases of AC; some of them were already being treated conservatively. Accordingly, the degree of LOM of the participants encompassed a rather wide range [23]. Because the objective of the study was more focused on evaluating diagnostic performance rather than comparing or demonstrating outcomes, the cutoff for LOM was set low to include various stages of AC.

Diagnostic performance for AC using Kinect depends on the cutoff values. We obtained cutoff values by three different ways (same as goniometric passive ROM cutoffs, reflecting mean differences, and optimal values from the ROC curves). Among them, the cutoff values from the ROC curves (flexion <158.3°, abduction <159.1°, or external rotation <59.1° [12]) showed the highest Cohen’s kappa values. Although the cutoff values from the ROC curves showed excellent performance, it is generally difficult to apply because these values depend on the current study population. Further research with more patients is required to investigate cutoff values that are acceptable and suitable for testing.

## Conclusion

In conclusion, the Kinect can be used to measure shoulder ROM based on its agreement with goniometric measurements and was clinically applicable to diagnose AC. The Kinect is an easy-to-use and nonintrusive device. We expect that the Kinect can be used for the clinical evaluation of the shoulder in practice for various conditions and purposes in the near future. For that purpose, further research is warranted to examine its clinical reliability in more sophisticated way and to evaluate its practical benefit.
